# Burnout, depression and suicidal ideation in dental students

**DOI:** 10.4317/medoral.19281

**Published:** 2013-10-13

**Authors:** Fernando Galán, José V. Ríos-Santos, Juan Polo, Blanca Rios-Carrasco, Pedro Bullón

**Affiliations:** 1 Department of Medicine, School of Medicine, University of Seville, Seville, Spain; 2 Department of Stomatology, Faculty of Dentistry, University of Seville, Seville, Spain; 3 Department of Preventive Medicine and Public Health, School of Medicine, University of Seville, Seville, Spain

## Abstract

Objectives: To investigate the prevalence, gender influence, and relationships between burnout, depression and suicidal ideation within the last year among second, fourth and fifth-year dental students. Study Design: A cross-sectional study was carried out in 212 dental students enrolled in the second, fourth and fifth years at the School of Dentistry of Seville using the Maslach Burnout Inventory-Student Survey and the MBI-Human Services Survey, the “Patient Health Questionnaire-2”, and the “Questions about Suicidal Ideation and Attempted Suicide”. Results: The response rate among dental students was 80%. Burnout prevalence in dental students was higher in second and fourth years than in fifth year (*p*= 0.059 and *p*= 0.003, respectively). Depression prevalence in the fourth year approached significance (*p*= 0.051). Prevalence of suicidal ideation within the past year was higher, yet not reaching significance, in fourth year. No gender-related differences were found. A significant association was observed between burnout and depression, and between depression and suicidal ideation (*p*< 0.001), but no association was found between burnout and suicidal ideation. Conclusions: This study has brought our attention to the high prevalence of burnout and depression, and reported for the first time the prevalence of suicidal ideation among dental students in preclinical and clinical years.

** Key words:**Burnout, depression, suicidal ideation, dental students.

## Introduction

Dentists seem to be prone to professional burnout, anxiety disorders and clinical depression because of the variety of sources of stress encountered throughout the professional career, and there is the possibility of beginning as early as university (1-4).

The term burnout was introduced in the 1970s by Maslach and Leiter (5), and was defined as a syndrome with emotional exhaustion (EE), depersonalization (DP) and diminished personal accomplishment (PA) that can occur among individuals working with people.

Research of dental student burnout has increased in recent years and most studies have been focused on clinical years (6-9). In these studies, the standard instrument used for burnout assessment was the Maslach Burnout Inventory-Human Services Survey (MBI-HSS), and the results were presented separately for each dimension of burnout. These were the reported values: high scores on EE between 10 and 39%, high scores on DP between 22 and 30%, and low scores on PA between 17 and 41 %. These results show a wide range depending on the cutoffs used and curricula of different dental schools studied, among other factors.

However, only one study has been focused on preclinical years (10). MBI-HSS was the instrument used and only the EE scale could be administered, and a wide variation between schools in high EE scores was observed, ranging from 3 to 26% (total 22%). “The DP and PA scales were not analyzed because the scale contains a number of specific points of contact with the patient, that a substantial proportion of the sample had not experienced” (10). Given the difficulties found to measure burnout in preclinical dental students using MBI-HSS, the application of the Maslach Burnout Inventory-Student Survey (MBI-SS) to study the risk of burnout in preclinical training years was suggested in our previous study, because it overcomes the difficulties due to students little or no previous contact with patients (11).

Among the demographic factors associated with burnout in dental students, gender showed conflicting results in the EE dimension of burnout on clinical years: females were significantly more emotionally exhausted than males (7-9). However, the opposite and no gender differences have also been reported (6,7).

Depression may be a consequence of prolonged burnout experience (12). Little attention has been paid to depression among dental students. The results of studies conducted on clinical years ranged between 2.8 and 15%, and between 7.4 and 14% on preclinical years (9,13,14), using different instruments and cutoff values to screen for depression. Considering gender, the abovementioned authors reported that more female than male dental students showed positive results for depression in clinical and preclinical years, except for Takayama (2), who found no gender differences among students in preclinical years.

To the best of our knowledge, there is no study on suicidal ideation among dental students, or its relationship with burnout and depression. A recent large, multi-institutional study using a mixed longitudinal and cross-sectional design was aimed at evaluating the prevalence of suicidal ideation among USA medical students as well as the relationship between suicidal ideation, burnout, and depression symptoms. In this study 49.6 and 11.2% were reported to have experienced burnout and suicidal ideation within the past year, respectively. Suicidal ideation in the previous year was significantly associated with burnout, and depressive symptoms (15).

The scarcity of studies on the prevalence of burnout and depression in dental students in preclinical years, conflicting results on the influence of gender on the prevalence of these variables, and the lack of research on suicidal ideation and its relation to burnout and depression among dental students led us to conduct this study.

The aims of this cross-sectional study were: 1) researching the prevalence of burnout, depression, and suicidal ideation within the past year in dental second-year students (preclinical training period) and in the fourth and fifth years (clinical training period), as well as whether their prevalence is influenced by gender, and 2) assessing the association between burnout, depression and suicidal ideation in this sample of dental students.

## Material and Methods

Participants

All dental students in second (final year of preclinical training) and fourth and fifth years (two final years of clinical training) enrolled in the School of Dentistry of Seville were invited to participate in this study. Data collection took place during an ordinary class in each course in midwinter semester in 2009. The lecturer explained the aims of the study and asked students for their cooperation. Then, questionnaires were distributed and collected after completion. Participation was voluntary and anonymous. Limited demographic information was obtained to ensure the confidentiality of the respondents and encourage participation and honest reporting. The study was approved by the ethical committee of the University of Seville.

Measurement of burnout

Burnout was assessed in second year dental students using the modified Spanish version of the Maslach Burnout Inventory General Survey (MBI-GS) (16), slightly adapted for university students, under the name of MBI-SS. The translated and validated Spanish version of the MBI-SS was applied (11,17). MBI-SS consists of 15 items that constitute three subscales: exhaustion (EX; 5 items), cynicism (CY; 4 items), and efficacy (EF; 6 items). All items are scored on a 7-point frequency rating scale ranging from 0 (never) to 6 (every day). According to normative data (18) the cut-off in exhaustion subscale (EX) is considered low if the score is 1.2 or less and high if it is 2.8 or higher, CY ≤ 0.6 and ≥ 2.25, EF ≤ 3.84 and ≥ 5.16 (11). Burnout was defined in this study as a high score in the exhaustion or cynicism subscales (11).

The MBI-HSS was used for fourth and fifth year dental students (19,20). This questionnaire consists of 22 items in three subscales: EE (9 items), DP (5 items), and PA (8 items). All items are scored on a 7-point frequency rating scale ranging from 0 (never) to 6 (every day). According to normative data from a Spanish sample comprising 1.138 people, included in the manual of the questionnaire (20). Cut-off in the EE subscale is considered low if the score is 15 or less and high if it is 24 or higher; on DP they are considered low if ≤ 4 and high if ≥ 9, and on PA, they are considered low if ≤ 33 and high if ≥ 39 (20). Burnout was defined for our study as a high score in the EE or DP subscales (15,20).

Screening for depression

Screening for depression was assessed by using the Patient Health Questionnaire-2 (PHQ-2). PHQ-2 includes the first 2 items of PHQ-9. The stem question is: “Over the last two weeks, how often have you been bothered by any of the following problems?” Both items are “little interest or pleasure in doing things” and “feeling down, depressed, or hopeless”. The response options for each item are “not at all,” “several days,” “more than half the days”, and “nearly every day”, which scored as 0, 1, 2, and 3, respectively. Thus, the PHQ-2 score can range from 0 to 6. PHQ-2 score ≥ 3 has 83% sensitivity, 90% specificity, and a positive likelihood ratio of 2.9 for major depression. Receiver operating characteristic (ROC) showed that the area under the curve (AUC) for PHQ-2 was 0.93 in the diagnosis of major depressive disorder and 0.90 in the diagnosis of any depressive disorder, compared to a structured psychiatric interview by independent mental health professionals (considering the standard criteria) (21).

Suicidal Ideation

Suicidal ideation was assessed by asking students: “During the past twelve months, have you had thoughts of taking your own life, even if you would not actually do it?”, and “During the past twelve months, have you made any attempt to take your own life?” These questions were taken from “Questions about Suicidal Ideation and Attempted Suicide” (15,22).

Statistical analysis

The internal consistency of the three subscales was assessed using Cronbach’s alpha. Descriptive statistics were used to estimate the prevalence of burnout, positive depression screen and suicidal ideation in dental students. The comparison of categorical variables such as gender and years of training was conducted using Pearson’s Chi-square or Fisher’s exact test. The level of statistical significance was set at or below 0.05. All analyses were performed using SPSS, v. 18.0.

## Results

Out of the 264 dental students enrolled at the School of Dentistry of Seville – (90 second year, 73 fourth year, and 101 fifth year students ) - 212 completed and returned their questionnaires, reaching a response rate of 80%. Four of them were excluded because no age or gender was reported. Therefore, only 208 were considered valid questionnaires, representing a response rate of 78.8%. Response rates were 83.4% (75 out of 90) among second-year students, 75.3% (55 out of 75) among fourth-year students, and 77.2% (78 out of 101) among fifth-year students. The age and gender of all students enrolled in the second, fourth and fifth years was very similar to that shown by respondents. All were white Caucasian. Demographic features are shown in Table 1. Internal consistency (Cronbach’s alpha) for the three subscales in the MBI-SS used in the second-year sample was: exhaustion 0.90, cynicism 0.76, and efficacy 0.73. And for the three subscales in the MBI-HSS, used in the fourth- and fifth-year samples internal consistency was: EE 0.88, DP 0.76, and PA 0.81.

**Table 1:**
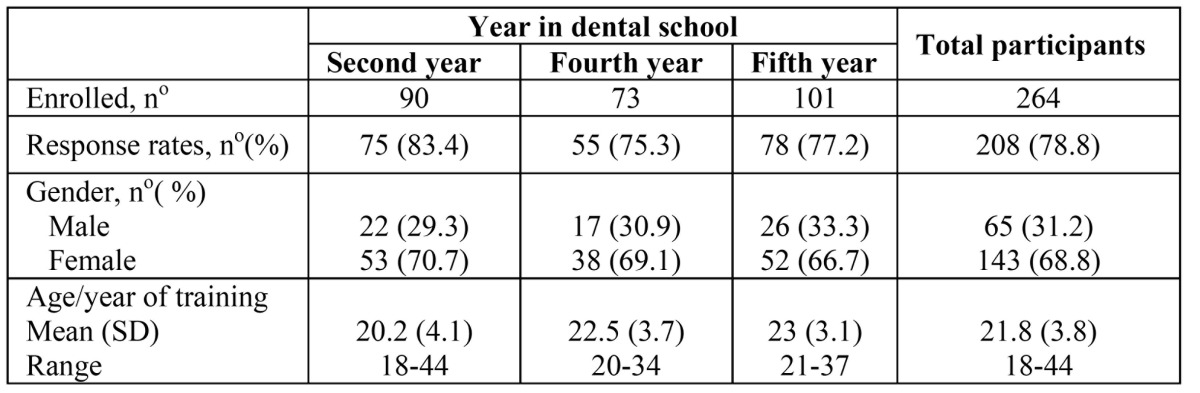
Characteristics of participants. Dental students enrolled, rates of valid response to the questionnaires, gender and age ratios (mean, SD, and range) per year at the School of Dentistry of Seville in academic year 2009-10.

Burnout, depression and suicidal ideation according to years of dental training 

Second and fourth year dental students showed significantly greater EE than their fifth-year mates (*p* = 0.019, and *p* < 0.001, respectively). In addition, fourth-year students showed significantly lower PA than second and fifth-year students (*p* = 0.003, and *p* = 0.05, respectively). No differences were found in the DP /cynicism subscale in any of the training years studied, as shown in  Table 2.

**Table 2:**
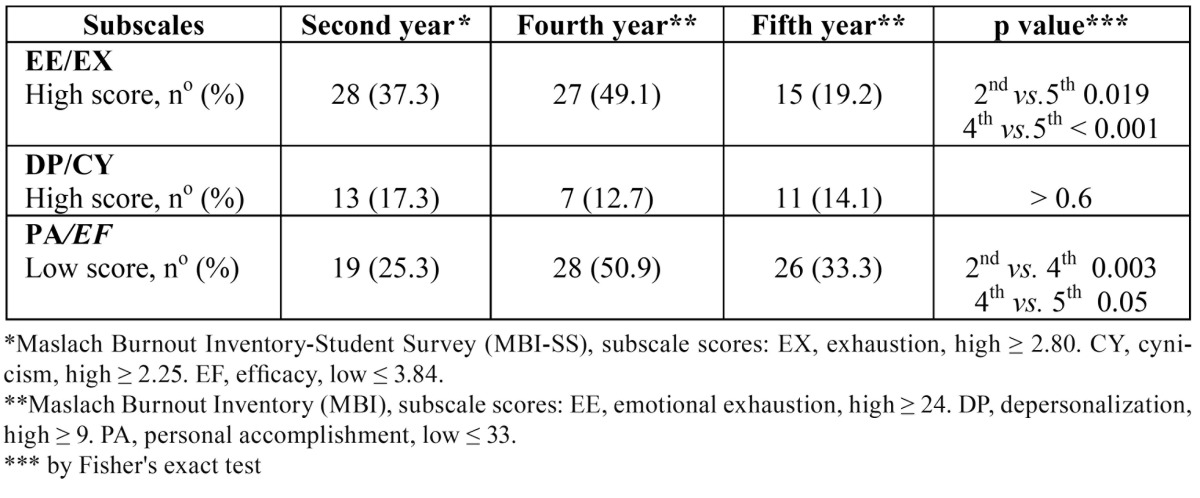
Number and percentage of high scores in emotional exhaustion/exhaustion, depersonalization/cynicism and low personal accomplishment/efficacy in second, fourth and fifth-year students from the School of Dentistry of Seville.

The prevalence of burnout in second- and fourth- year dental students was higher than in fifth- year ones, reaching significance in fourth- year (*p* = 0.003), yet close to significance levels in second- year students (*p* = 0.059). 

The prevalence of depression in the second and fourth years was higher than in the fifth year, yet very-close-to-significance levels were only observed in fourth-year students relative to their fifth- year mates (*p* = 0.051).

The prevalence of suicidal ideation in second- and fourth- year dental students was higher than in their fifth- year mates, but no significant differences were found. The results are shown in  Table 3.

**Table 3:**
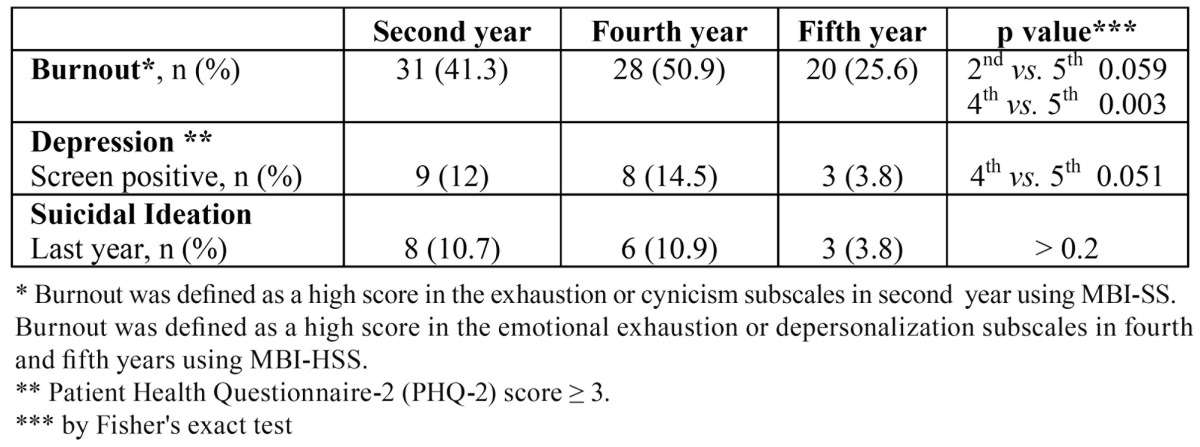
Number and percentage of burnout, positive screen depression, and suicidal ideation by year at the School of Dentistry of Seville.

Gender was observed to have no significant influence on the prevalence of burnout, depression, and suicidal ideation (*p* > 0.60)

Furthermore, only 2 (1%) of the second and fourth year participants reported a suicide attempt within the last year.

Association between burnout, depression and suicidal ideation in the general sample of dental students

The association between burnout and a positive screen for depression was highly significant (*p* < 0.001). The association between burnout and suicidal ideation within the previous year was not significant (*p* = 0.07). The association between students with a positive screen for depression and suicidal ideation in the previous year was highly significant (*p* < 0.001).

## Discussion

Our study has several limitations. Firstly, this study is cross-sectional and cannot therefore determine causal relationships. Secondly, although the response rate among dental students was above 75%, there were non-responders students that may well bias it. Burnout students can be hypothesized to be either less motivated to fill out a survey or more likely to participate because the topic is relevant to them. Thirdly, the small number of students with depression and suicidal ideation may affect statistical analysis results on the relationship among variables. And, finally, since this study is based on a single institution, the results might not be representative of all dental undergraduates in Spain.

Our study has several important strengths. Firstly, it is the first study on the prevalence of burnout and depression in dental students in Spain. Secondly, our study on the prevalence of suicidal ideation within the past year among dental students had not been previously reported in literature. Thirdly, validated metrics were used to measure burnout and depressive symptoms, which allowed comparison with both the general population and other samples such as medical students. Fourthly, we asked about suicidal ideation by using questions from an already existing inventory (22) that had previously been used to assess suicidal ideation among medical students (15).

The main reason for using the MBI-SS was the features of our student population. Specifically, MBI-SS allows overcoming the difficulties faced when students have no contact with patients, as it is the case for second-year students, who belong to the preclinical period (11).

When conducting research on burnout, it may be difficult to decide whether one should report results separately - for each burnout dimension - or whether one should combine the dimensions. The last option may be useful for researchers who wish to estimate the prevalence of burnout in a sample (23). The criteria used to define burnout in our previous study (11) and the present research - combining dimensions - were as follow: burnout was defined as a high score on the subscales of exhaustion or cynicism in the preclinical years using MBI-SS, and as a high score on the EE or DP subscales in clinical years by MBI-HSS, like other studies on burnout (7,15).

Direct comparisons between our results with those reported in literature should be made with caution due to the variety of cutoffs used for burnout subscales, the varied criteria used to define burnout, the different scales used to measure depression and, finally, the curricular differences among dental schools.

In our study, preclinical second- year students showed higher levels of EE than those reported by Humphris (10) in their study on first-year students from seven European dental schools. However, if the results of Cork and Greifswald schools - which follow a similar syllabus to ours and whose students had no contact with patients either - are observed in greater detail, the average percentage of students with high scores on EE was similar (37.3% vs 37%). Burnout prevalence was also found high in our study; this may be explained by the fact that lab-practice doubled in the second year, but the number of theoretical credits remained unchanged, compared to first year students ( Table 4).

**Table 4:**
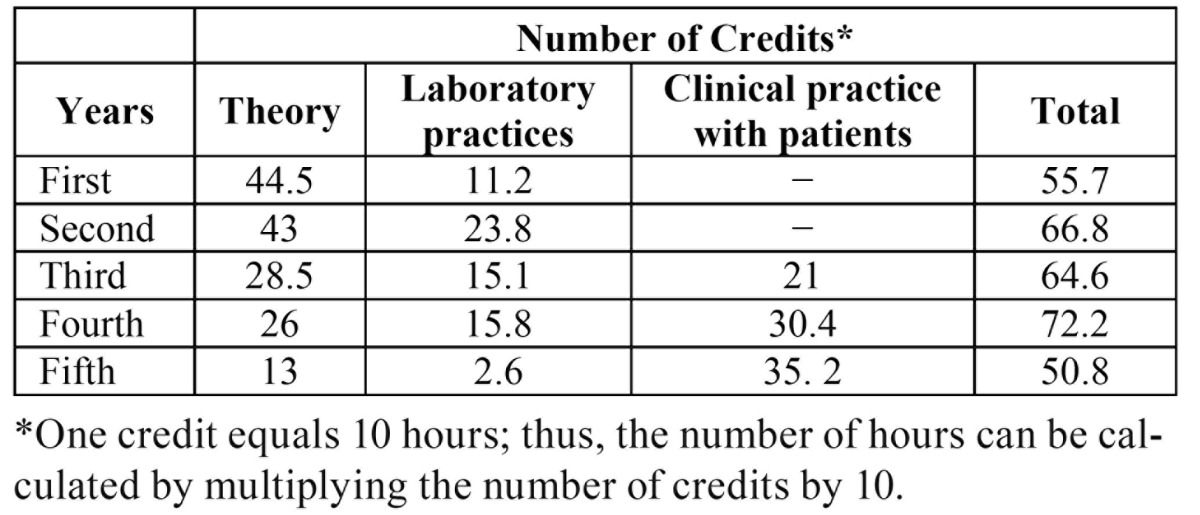
Distribution of theoretical and practical number of credits per year at the School of Dentistry of Seville in 2009-10.

In addition, our study on the clinical years found that fourth- year students showed significantly higher levels of EE and significantly lower levels of PA - along with burnout levels that double those found in fifth year students. Our results are slightly different from those reported in literature. Pöhlmann (6) found no difference between fourth and fifth year the dental students. Gorter (7) reported results in fifth year dental students, who followed a similar trend to ours fourth year students. However, a recent study conducted by Prinz (9) on dental students in the fourth and fifth academic years, reported quite similar findings to ours. Higher levels of burnout in fourth than in fifth year dental students, may be partly explained by the fact that the former are those with the greatest academic overload (72.2 credits). In addition, the latter had a 30% reduction of total credits (50% in theoretical contents, and 83% in lab practices, while clinical practices with patients increased 14% relative to the fourth- year ( Table 4).

Lower prevalence of depression than reported in a longitudinal study by Newbury-Birch (13) on second- and fifth- year students was found in the present study. Like us, they found no differences between both years. In addition, our results are consistent with the findings reported in a recent 3-year longitudinal study (14). At follow-up showed a significant increase from fourth to fifth year and then a significant decrease in the sixth year. It should be borne in mind that clinical training begins in the fifth year in this dental school. Some curricular aspects may partly explain our results. The highlights of the second year were: a 48% increase in lab practices and the lack of patient contact, and the highlight in the fourth year seemed the greatest academic burden among all years. The clear decrease in the prevalence of depression among fifth year students may be explained by the reduction in total academic load and perhaps improved skills in clinical practice, and even the fact of being closer to graduation.

Among demographic factors associated to burnout and its subscales, or depression in dental students, gender shows conflicting results (6-9). In our study, gender had no significant influence on burnout - or any of its subscales -, nor on depression. These negative results may be explained by the fact that most participants were women (68.8%), which adequately reflects gender trends at dental schools over the last decades. Perhaps this recent trend has alleviated some of the pressures previously experienced by women to equal and even outperform their male counterparts so as to prove their worth in then male-dominated fields (7,11).

The prevalence of suicidal ideation in second and fourth-year dental students was higher than in their fifth year counterparts, but no significant differences were found. In the overall sample, suicidal ideation in the last year was reported by 17 (8.2%) out of the 208 valid respondents. Assuming all nonresponders had no suicidal ideation, the prevalence of suicidal ideation in the past 12 months would be 6.4% for all 264 students. These results are similar to those reported in a recent study conducted on students from seven medical schools in the USA: 11.2 and 5.8%, respectively (15). Like them, we found no influence of gender. Of course, they are not fully comparable because a larger sample of medical students was examined in their study.

Our results on suicidal ideation in dental students cannot be compared with other studies, since there is no other study on suicidal ideation in literature. No study on 1-year prevalence of suicidal ideation in people of similar age in the general population of Spain (20 to 24-year-olds) has been published to date. However, a cross-national research studying the 2-week prevalence of suicidal ideation on general population was conducted in five European countries, including Spain (24). In the sample from Spain, within the range of 15-29 year-olds, the prevalence of suicidal ideation was 2.3%, and were not reported suicide attempts or the influence of gender.

The strong association found between burnout and depression, and between depression and suicidal ideation in the previous year is consistent to those reported in medical students by Dyrbye (15). Unlike them, we found no association between burnout and suicidal ideation in the previous year. This may be partly due to the small number of suicidal ideation.

In conclusion, the results of our cross-sectional study showed that dental students in the second and fourth year doubled the prevalence of burnout, and also tripled the prevalence of depression and suicidal ideation in relation to fifth- year students, and that gender had no influence. In addition, a strong association was found between burnout and depression and between depression and suicidal ideation, but not between burnout and suicidal ideation. Among other factors, academic overload may contribute to differences in burnout, depression and suicidal ideation among dental students. However, coping mechanisms should also be taken into account. A recent study by Prinz (9) showed that dental students with high levels of burnout and depression simultaneously showed high levels of dysfunctional coping.

Our findings may also have important implications for the determination of the most appropriate age and training years to start the detection and prevention of student distress and suicidal ideation. We propose an intervention that may include reconsidering the traditional curriculum, individual career counseling, education on prevention and stress management, and coping resources, providing students with information on confidential resources available on their own or off-campus institution covered by student health insurance.

However, further studies - which will be more valuable if carried out longitudinally - are needed to confirm these findings.
